# Postexposure Antimicrobial Drug Therapy in Goats Infected with *Burkholderia pseudomallei*

**DOI:** 10.3201/eid3105.241274

**Published:** 2025-05

**Authors:** Richard A. Bowen, Airn E. Hartwig, Angela M. Bosco-Lauth, Josilene N. Seixas, Jana M. Ritter, Pamela S. Fair, Mindy G. Elrod, Zachary P. Weiner, Robyn A. Stoddard, Antonio R. Vieira, Rachel M. Maison, Elizabeth Lawrence, Hannah Sueper, Mckinzee Barker, William A. Bower

**Affiliations:** Colorado State University College of Veterinary Medicine and Biomedical Sciences, Fort Collins, Colorado, USA (R.A. Bowen, A.E. Hartwig, A.M. Bosco-Lauth, R.M. Maison, E. Lawrence, H. Sueper, M. Barker); Centers for Disease Control and Prevention, Atlanta, Georgia, USA (J.N. Seixas, J.M. Ritter, P.S. Fair, M.G. Elrod, Z.P. Weiner, R.A. Stoddard, A.R. Vieira, W.A. Bower)

**Keywords:** Melioidosis, *Burkholderia pseudomallei*, antimicrobial resistance, prophylaxis, goats, bacteria, postexposure prophylaxis

## Abstract

Infection with *Burkholderia pseudomallei*, the causative agent of melioidosis, occurs by exposure to the organism in soil or water. There is concern for *B. pseudomallei* use as a potential bioweapon and as an exposure hazard in diagnostic laboratories processing samples or cultures containing the bacterium. The optimal strategies for treatment and postexposure prophylaxis are inadequately developed. This study used goats to evaluate 3 antimicrobial drug treatment regimens for postexposure therapy because they are a species naturally susceptible to *B. pseudomallei* infection. Goats were infected by percutaneous inoculation, and antimicrobial drug therapies were initiated 48 hours later. Widespread infection with abscess formation in multiple organs developed in untreated goats and goats treated with either amoxicillin/clavulanate or sulfamethoxazole/trimethoprim. In contrast, treatment with the combination of all 4 antimicrobial drugs might have eradicated the infection. Our findings suggest combination therapy with those 4 antimicrobial drugs may be useful for postexposure prophylaxis in humans.

Melioidosis is an infectious disease caused by the soilborne saprophytic gram-negative bacterium *Burkholderia pseudomallei* (*1*–*3*). The organism is endemic in large regions of southeast Asia and northern Australia (*4*) and has been detected in the Caribbean (*5*), South America (*6*), and most recently in the gulf coast region of the United States (*7*,*8*).

*B. pseudomallei* is a public health concern and has a very broad host range, causing disease in many domestic and wild mammals and even ectothermic vertebrates (*9*–*11*). Although a wide range of animal species are susceptible to infection, melioidosis is not typically considered a zoonotic disease. However, animals can shed *B. pseudomallei* in the environment, and therefore, infected animals are a potential source for human transmission (*12*,*13*).

In addition to the large burden of naturally occurring melioidosis, there are 2 additional causes of concern. First, *B. pseudomallei* is a potential bioweapon and is classified as a tier 1 select agent by the US government because of its low infectious dose by inhalation and resistance to conventional antimicrobial therapy. Second, there are concerns for accidental exposure of clinical or research laboratory personnel by needle stick or aerosol, especially when isolation of *B. pseudomallei* is not expected and biosafety practices are inadequate (*14*–*16*). In contrast to natural disease, where exposure time is likely unknown and could have occurred many weeks if not years earlier, the timing of deliberate or accidental exposures could be known, providing the opportunity for rapid postexposure prophylaxis (PEP). Those scenarios highlight the need to determine the most effective treatment regimen for PEP.

Clinical manifestations of melioidosis are highly variable in both humans and animals and may involve abscess formation in multiple organs, pneumonia, cutaneous lesions, and sepsis (*12*,*17*). Guidelines for therapy in humans are in place and widely used in endemic regions on the basis of long-standing clinical experience (*18*). A major challenge in the treatment of melioidosis is that *B. pseudomallei* is intrinsically resistant to many antimicrobial drugs, and eradication usually involves prolonged therapy, often with >1 antimicrobial agent (*19*,*20*). Melioidosis therapies in animal models are poorly studied and only in the context of acute PEP in mice (*21*–*23*). The common finding from those mouse studies is that antimicrobial drug therapy must be initiated rapidly after the inoculation of *B. pseudomallei*, and although critical extension in postinoculation survival can be attained, the organism is not eliminated. The goal of this article was to evaluate 2 commonly used antimicrobial drug treatment regimens, alone and in combination, for postexposure therapy of *B. pseudomallei* infection in goats, a natural host model for melioidosis.

## Materials and Methods

We conducted all animal studies in compliance with the Animal Welfare Act and as approved by the Colorado State University Institutional Animal Care and Use Committee, the Institutional Biosafety Committee, and with approval from the Federal Select Agent Program. We conducted all studies under Biosafety Level 3 (BSL-3) or Animal BSL 3 (ABSL-3) containment at Colorado State University. The number of animals per treatment group was determined by available ABSL-3 space.

### Bacterial Strain

The strain of *B. pseudomallei* we used to inoculate study goats was an isolate from an infected goat in Australia designated Bp 4176/MSHR 511 (*24*) and was originally provided to us by Dr. Apichai Tuanyok (University of Florida, Gainesville, Florida, USA). We cultured the bacteria for inoculation in Muller-Hinton broth at 37°C in air with constant shaking, harvested at the mid-log phase of growth, and stocks containing 15% glycerol were stored at −80°C.

### Culture Methods

For tissues with grossly visible abscesses, we excised the samples collected for bacterial culture away from major gross lesions. For bacterial isolation, we collected ≈100 mg samples from tissues into homogenizing tubes containing 0.9 ml of brain–heart infusion broth supplemented with 10% glycerol. We homogenized those samples and then froze them to −80°C until processing.

We thawed, vortexed, and briefly centrifuged to pellet tissue debris (2,000 × *g* for 10 s) of the frozen tissue homogenates, spread 0.1 mL of each sample onto a 10 cm Ashdown’s agar plate, and incubated the plates at 37°C. We examined the plates 48 hours after inoculation and performed colony counts. We sampled representative colonies that appeared to be *B. pseudomallei* on the basis of morphology and color, along with colonies that did not appear to be *B. pseudomallei* and used them to prepare spot slides that were fixed for 15 minutes with 80% acetone. We immunostained those slides along with known positive and negative (*Escherichia coli*) slides by using an antibody to *B. pseudomallei* capsular polysaccharide (*25*) to confirm their identity.

### Antimicrobial Drugs

We conducted a preliminary pharmacokinetic study to confirm blood concentrations of 3 of the 4 drugs after oral administration to 2 goats from another project. ELISA kits for trimethoprim, sulfamethoxazole, and amoxicillin were purchased from MyBioSource (https://www.mybiosource.com), and we assayed serum samples according to the manufacturer’s instructions. Each kit contained standards that we used to prepare standard curves. We did not assay serum for concentrations of clavulanate.

### Animals, Challenge Procedures, and Clinical Observations

We purchased young adult female goats from a private source and clinically evaluated them to ensure baseline health; they were weighed several days before challenge and had an average weight of 70 kg (63–82 kg). We implanted a Biothermo-Lifechip (Destron-Fearing, https://www.destronfearing.com) subcutaneously in each goat for identification and easy monitoring of body temperature. We housed the goats by group (8 animals per 12- × 18-foot room) under ABSL-3 containment for the duration of the study. We fed the goats alfalfa hay supplemented with grain.

We performed percutaneous challenge by a combination of subcutaneous and intradermal injection over the shoulder region, with a target dose of 10^4^ CFU in 0.2 mL of solution. We diluted the bacteria in phosphate buffered saline from frozen-thawed stocks. We evaluated the goats clinically 2 times daily for the duration of the study. We recorded the goat’s body temperature from their microchip 2 times daily for the first 10 days, then 1 time daily until euthanasia.

We evaluated 4 antimicrobial drug therapies, each in 8 goats: 1, no treatment; 2, amoxicillin/clavulanate; 3, sulfamethoxazole/trimethoprim; and 4, a combination of amoxicillin/clavulanate and sulfamethoxazole/trimethoprim. We initiated drug treatment 48 hours postchallenge; treatment consisted of oral gavage of 25 mL with a dosing syringe. We prepared drugs by dissolving the requisite number of tablets in deionized water within 30 minutes of treatment. Both types of tablets were manufactured by Aurobindo Pharma (https://www.aurobindo.com) and provided in the following formulations: amoxicillin/clavulanate, 500/125 mg/tablet; sulfamethoxazole/trimethoprim 1,200/240 mg/tablet. On the basis of tablet composition, the goats received the doses and approximate dosages of drugs 2 times daily for 21 days, ceasing on day 23 postchallenge (Table 1).

**Table 1:** Antimicrobial drug treatments administered to goats in evaluation of postexposure antimicrobial drug therapy in goats infected with *Burkholderia pseudomallei*

Group	Amoxicillin	Clavulanate	Sulfamethoxazole	Trimethoprim
1	None	None	None	None
2	500 mg, ≈7.1 mg/kg	125 mg, ≈1.8 mg/kg	None	None
3	None	None	1200 mg, ≈17.1 mg/kg	240 mg, ≈3.4 mg/kg
4	500 mg, ≈7.1 mg/kg	125 mg, ≈1.8 mg/kg	1200 mg, ≈17.1 mg/kg	240 mg, ≈3.4 mg/kg

### Serologic Analyses

We assayed serum collected before and after challenge for antibodies by using 2 techniques. The indirect hemagglutination assay was performed as described previously (*26*). For whole cell lysate ELISA, we used a lysate of *B. pseudomallei* Bp82 that we prepared by using techniques similar to those previously described (*27*,*28*). We coated plates with a solution containing 3 µg/mL of lysate, blocked with phosphate-buffered saline containing dried skim milk (blocking buffer), exposed to the test serum diluted 1:100 in blocking buffer, washed again, and then exposed to a horseradish peroxidase-protein A/G conjugate. After a final washing, we added ABTS substrate and, after stopping the reaction, we read absorbance at 450 nm. The cutoff for positivity was set at 3 SDs above the mean value for all preinoculation serum.

### Postmortem Analyses and Radiography

We euthanized goats at 14–28 days postinfection (dpi) (Appendix Table 1) by intravenous injection of an overdose of pentobarbital. We then performed collection and gross examination of spleen, lungs, liver, lymph nodes (mandibular, mesenteric, mediastinal, retropharyngeal, prescapular), kidney, and skin. We recorded the occurrence of visible abscesses in those tissues and the extirpated lungs were radiographed. We fixed tissue samples in 10% neutral buffered formalin.

### Histopathology and Immunohistochemistry

We processed formalin-fixed tissues by routine paraffin histology. We sent tissue blocks to Center for Disease Control and Prevention’s Infectious Diseases Pathology Branch (Division of High-Consequence Pathogens and Pathology, National Center for Emerging and Zoonotic Infectious Diseases), where they were sectioned at 4 µm, mounted on glass slides, and stained with hematoxylin-eosin for histopathologic evaluation. Two veterinary pathologists visually assessed the slides for the presence of inflammatory or other lesions and for *B. pseudomallei* immunoreactivity.

We conducted immunohistochemical (IHC) assays on select tissues on the basis of 2 criteria: the presence of lesions consistent with *B. pseudomallei* infection observed grossly or microscopically; or tissues with positive result for *B. pseudomallei* on bacterial culture. We tested similar types and numbers of tissues from each treatment group. We also examined a subset of tissues from animals without gross or microscopic lesions or with negative cultures. For the detection of bacterial antigen, we used a rabbit polyclonal *B. pseudomallei* antibody at 1:1,000 dilution. We performed colorimetric detection of attached antibodies by using the Mach 4 AP polymer kit (Biocare Medical, https://biocare.net) at room temperature and with heat-induced epitope retrieval. Using EDTA buffer, we conducted heat-induced epitope retrieval by using the NxGen decloaker (Biocare Medical) at 110°C for 15 minutes. We blocked all slides in Background Punisher (Biocare Medical) for 10 minutes and incubated with primary antibody for 30 minutes. We applied Mach 4 Polymer for 30 minutes (Biocare Medical) and visualized the antibody polymer conjugate by applying fast red chromogen dissolved in naphthol phosphate substrate buffer (Sigma Aldrich, https://www.sigmaaldrich.com) to tissue sections for 30 minutes. We ran the negative control serum in parallel. We counterstained slides with Mayer’s hematoxylin (Poly Scientific, https://www.statlab.com) and blued in lithium carbonate (Poly Scientific). Positive controls included formalin-fixed, paraffin-embedded human tissue from a patient infected with *B. pseudomallei*.

### Statistical Analyses

We evaluated the differences among treatment groups in the number of animals with positive or negative outcomes for different parameters that were evaluated by pairwise contingency tables. We used Fisher exact test by using GraphPad Prism (https://www.graphpad.com) (Appendix Table 2).

## Results

### Preliminary Pharmacokinetic Study

**Figure 1:**
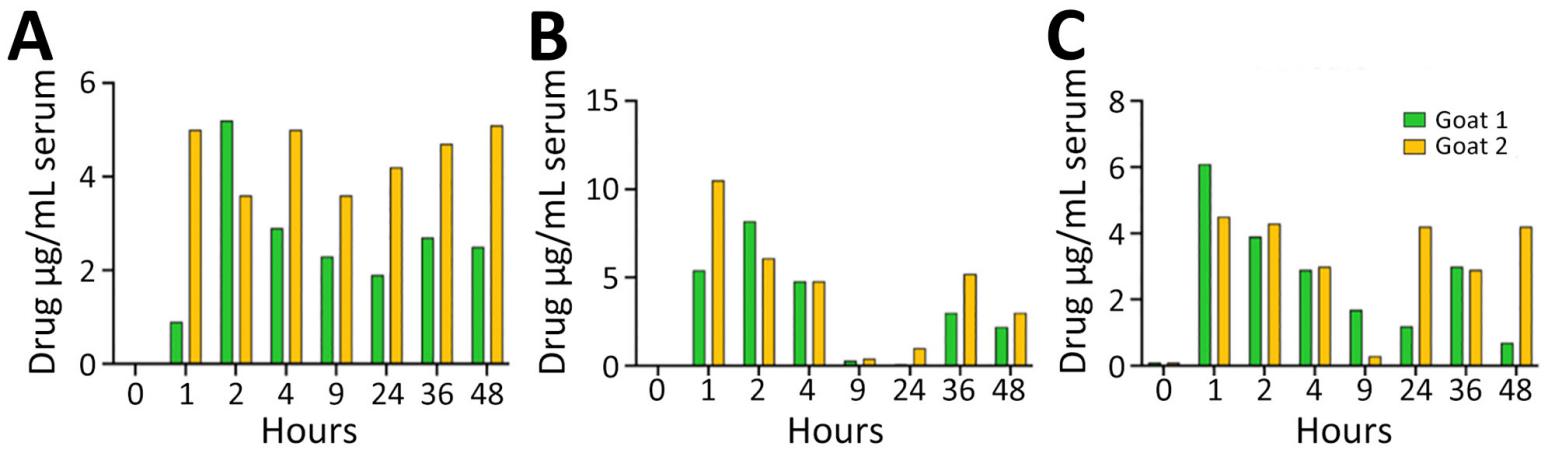
Pharmacokinetic analysis of antibiotics used to study postexposure antimicrobial drug therapy in goats infected with *Burkholderia pseudomallei*. Two female goats received a mixture of 1 capsule containing sulfamethoxazole (800 mg) and trimethoprim (160 mg) plus 1 capsule containing amoxicillin (500 mg) and clavulanate (125 mg) in a total of 25 mL of water. We repeated the treatment 12 hours later and collected blood samples at 1, 2, 4, 9, 24, 36, and 48 hours after the initial treatment. Concentrations of amoxicillin (A), sulfamethoxazole (B), and trimethoprim (C) over the 48-hour period showed acceptable blood levels of the drugs after oral administration.

We used 2 female goats for the antibiotic pharmacokinetic study; goat 1 weighed 64 kg and goat 2 weighed 33 kg. We treated the goats by using a dosing syringe with a mixture of 1 capsule containing sulfamethoxazole (800 mg) and trimethoprim (160 mg) plus 1 capsule containing amoxicillin (500 mg) and clavulanate (125 mg) in a total of 25 mL of water. We repeated the treatment 12 hours later. We collected blood samples at 1, 2, 4, 9, 24, 36, and 48 hours after the initial treatment and stored serum frozen until ELISA. We recorded the concentrations of amoxicillin, sulfamethoxazole, and trimethoprim over the 48-hour period (Figure 1). The treatments resulted in acceptable blood levels of the antimicrobial drugs after oral administration.

### Clinical Response to Infection

All goats remained clinically unremarkable through 5 dpi and after 3 days of treatment. By 7 days following initiation of treatment, most goats displayed signs of lethargy, anorexia, and diarrhea. This effect was likely because of a combination of infection and adverse influence of the antimicrobial drug therapy on ruminal microbiota. Because of the occurrence of severe disease and for humane considerations, we euthanized groups of goats beginning at 14 dpi. To maintain an ability to compare pathology among groups, we euthanized equal numbers of goats from each group at some of the euthanasia time points (Appendix Table 1).

### Gross Pathological Findings and Radiology

**Figure 2:**
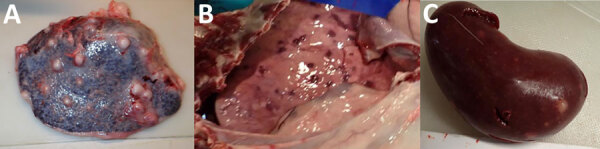
Examples of grossly visible postmortem lesions observed in goats infected with *Burkholderia pseudomallei* in study of postexposure antimicrobial drug therapy. A) Spleen of goat 8429, treated with amoxicillin/clavulanate; B) lung of goat 8549, not treated; C) kidney of goat 8430, treated with amoxicillin/clavulanate.

**Figure 3:**
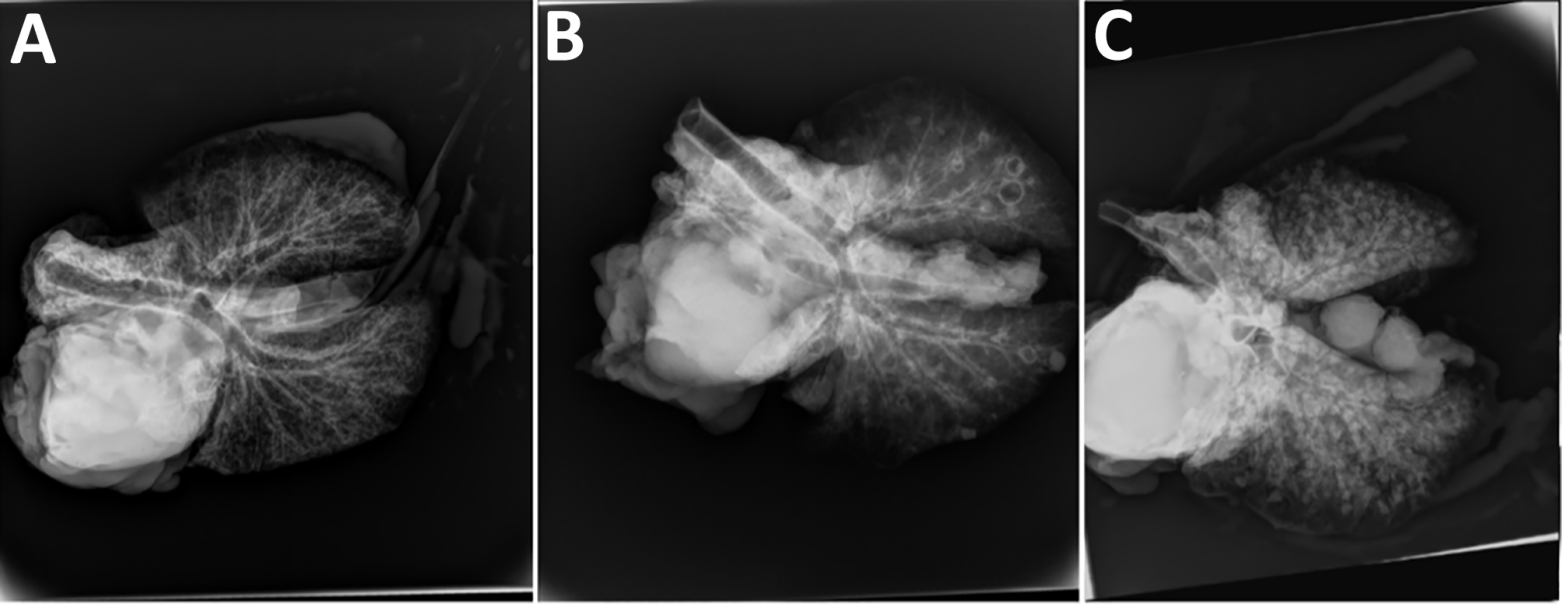
Examples of postmortem pulmonary lesions observed by using radiography in extirpated lungs of goats infected with *Burkholderia pseudomallei* in study of postexposure antimicrobial drug therapy. A) Goat 1197, not treated, showing no visible abscesses; B) goat 8430, treated with amoxicillin/clavulanate, showing moderate abscesses; C) goat 8549, not treated, showing severe abscesses.

The most common gross lesion we observed was splenic abscess, but abscesses were also detected in kidney, liver, and lung (Table 3; Figure 2). We radiographed extirpated lungs at necropsy to assist in assessing the magnitude of pulmonary abscessation. The number and size of pulmonary lesions varied considerably among goats (Figure 3) and generally corresponded with number of abscesses identified grossly.

### Bacterial Culture from Tissues

We individually homogenized and plated 10 tissues from each goat on Ashdown’s medium to determine whether viable *B. pseudomallei* was present. We compiled results of analyses for individual goats (Appendix Table 3) and by treatment group (Table 2). Treatment with the combination of all 4 antimicrobial drugs had a clear benefit in the number of tissues colonized with bacteria compared with no treatment or treatment with only 2 antimicrobial drugs (Appendix Table 2).

**Table 2:** Comparison of bacterial culture and abscesses observed at necropsy or by histologic evaluation of major organs by treatment group in evaluation of postexposure antimicrobial drug therapy in goats infected with Burkholderia pseudomallei*

Observation	Treatment			
	None	Amoxicillin/clavulanate	Sulfamethoxazole/trimethoprim	Sulfamethoxazole/trimethoprim + amoxicillin/clavulanate
No. goats with >1 positive *B. pseudomallei* tissue culture	6 of 8	8 of 8	4 of 8	0 of 8
Organs with macroscopic abscesses				
Spleen	8 of 8	8 of 8	5 of 8	0 of 8
Lungs	4 of 16	10 of 16	3 of 15	0 of 16
Liver	0 of 8	3 of 8	0 of 8	0 of 8
Kidney	0 of 8	3 of 8	0 of 8	0 of 8
Organs with microscopic abscesses				
Spleen	6 of 8	8 of 8	5 of 8	0 of 8
Lungs	3 of 16	11 of 16	0 of 15	0 of 16
Liver	1 of 8	3 of 8	0 of 8	0 of 8
Lymph nodes	5 of 8	8 of 8	2 of 8	0 of 8
Kidney	0 of 8	0 of 7	0 of 8	0 of 8

*Statistical analyses of differences between treatment groups are available (Appendix Table 2).

### Histopathology and Immunohistochemistry

**Figure 4:**
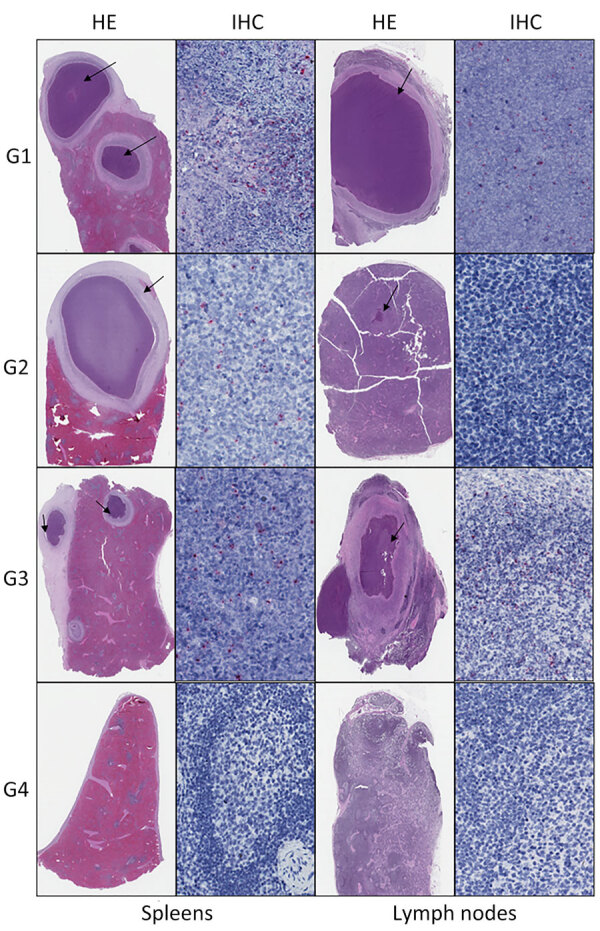
Histopathologic lesions (arrows) and immunocytochemical localization of *Burkholderia pseudomallei* in spleen and lymph nodes of goats infected with *Burkholderia pseudomallei* in study of postexposure antimicrobial drug therapy.

Abscess or other suppurative inflammation, including occasional pyogranulomas, were consistent histopathologic findings we observed in several organs among all treatment groups except group 4, the goats treated with amoxicillin/clavulanate and sulfamethoxazole/trimethoprim (Table 2). We often observed a typical abscess in experimental infection of *B. pseudomallei*, which is composed by an external fibrotic capsule with epithelioid macrophages within the intermediate layer and with a center containing neutrophils, cellular debris, and fibrin. Lesions were similar at 14 and 28 dpi. Focal or multifocal abscesses were most common in the spleen. We also observed acute and chronic inflammation of variable severity and distribution in a subset of all other examined organs (Table 2; Figure 4).

More acute lesions (14 dpi) had an inflammatory exudate composed of many viable and necrotic neutrophils, whereas more mature abscesses (28 dpi) had more pronounced fibrosis. After 14 dpi, we identified microscopic lesions in fewer animals (n = 4), but lesions were more numerous when found and mainly confined to the spleen; 1 goat at 14 dpi had an abscess in the mediastinal lymph node, and another goat had abscesses in the lungs and liver. We saw few multinucleated giant cells in lymph nodes (mesenteric and retropharyngeal lymph nodes) of 2 animals (from groups 2 and 4); however, we did not find granulomas in any animal.

Although abscesses were more numerous at later time points, the distribution and quantity of the immunostaining was similar among groups 1–3. In the group treated with amoxicillin/clavulanate and sulfamethoxazole/trimethoprim, we observed no gross or histopathologic lesions and no immunohistochemical evidence of *B. pseudomallei*. Immunohistochemical staining for *B. pseudomallei* was predominantly in the spleen and lymph nodes, with limited staining in other organs. Of 70 select tested tissues, including those with and without abscesses microscopically, 43 showed multifocal immunoreactivity by IHC at 14–28 dpi, which was strongly correlated with the presence of abscess. Of 40 tissues with histologic evidence of abscess (18 had no reported gross lesions and 15 were negative by culture), 38 showed multifocal immunostaining. No immunostaining was seen in 25 of 30 tissues without microscopic lesions; only 5 were immunoreactive. Of those 5 immunoreactive tissues, 2 had gross lesions and positive culture results, and 3 tissues had no gross lesions and the culture was negative; however, those tissues were from 2 animals (goat 8549, group control; goat 8637, group sulfamethoxazole/trimethoprim) who had abscesses and culture positive in other organs. The distribution of *B. pseudomallei* antigen was typically extracellular, within the necrotic center of the abscesses, and rarely in the cytoplasm of some apparently viable neutrophils (Figure 4).

Besides the abscesses or other neutrophilic infiltrates in different organs, we observed mild changes consisting of moderate-to-severe congestion, lymphoid hyperplasia, sinus histiocytosis, lymphoplasmacytic perivascular, or interstitial inflammation in a few cases. In addition, we observed nonspecific lesions such as hepatic steatosis and hyalinization of the follicular centers of spleens. Vasculitis was evident only in the liver of 1 case (goat 8434), euthanized at 23 dpi. We performed IHC on select tissues without gross or microscopic lesions or with negative cultures; we examined those tissues and observed no immunostaining.

### Serology

**Figure 5:**
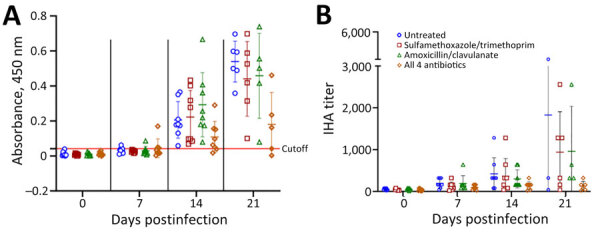
Serologic responses of goats to infection with *Burkholderia pseudomallei* in study of postexposure antimicrobial drug therapy. A) Responses measured by using whole-cell ELISA. Cutoff for positivity was set at 3 SDs above the mean value for all preinoculation serum. B) Responses measured by indirect hemagglutination assays. Horizontal lines indicate means; error bars indicate standard deviation.

According to whole-cell ELISA, all but 2 goats (8437 and 8555) had seroconverted by 21 dpi; the 2 goats that failed to seroconvert were in the group treated with the combination of all 4 antimicrobial drugs (Figure 5, panel A). The mean magnitude of antibody response was also significantly lower (p<0.05) in the animals receiving the combination of all 4 antimicrobial drugs. The indirect hemagglutination assay demonstrated the same trends as the whole cell lysate ELISA (Figure 5, panel B; Appendix Table 4).

## Discussion

The treatment of melioidosis presents substantial challenges because of the intrinsic resistance of *B. pseudomallei* to many antimicrobial drugs, the intracellular nature of the bacteria in infected patients, and the tendency for infections to become latent and recrudesce after treatment is discontinued. Most current recommendations for the treatment of melioidosis involve a biphasic regimen with several weeks of parenteral administration of antimicrobial drugs, followed by months of eradication therapy with orally administered antimicrobial drugs (*18*,*32*). In situations where exposures may have occurred, guidelines have been developed and internationally adopted for PEP, recommending trimethoprim/sulfamethoxazole for 21 days in high-risk exposures and for those with predisposing underlying conditions (diabetes, renal or liver disease, or other immune-suppressing conditions); PEP should be offered even after low-risk exposures (*33*). However, those recommendations are on the basis of limited mouse-model studies that demonstrated efficacy only if PEP was started within 48 hours of exposure, something that is not realistic. Goats are natural hosts for *B. pseudomallei* infection (*9*,*29*) and were shown to be highly susceptible to both percutaneous and aerosol exposure to *B. pseudomallei* (*30*,*31*). 

The objective of this study was to use the goat model to evaluate the efficacy of 3 antimicrobial drug regimens as PEP for *B. pseudomallei* infection induced by percutaneous inoculation. Overt clinical disease manifested in all the goats, including those with minimal lesions at necropsy after treatment with all 4 antimicrobial drugs. However, it was not clear if the observed clinical illness was related to infection with *B. pseudomallei* or adverse events related to therapy. The control animals in group 1 did not receive antimicrobial drugs and had more severe clinical signs and lesions. This increased severity suggests clinical disease in the other groups was likely a result of the infection plus disruptions of ruminal microbiota because of antimicrobial drug therapy, which is a disadvantage to this model and not likely to be a major issue in humans. Another shortcoming of this study is that the pharmacokinetic study we conducted with 2 goats was far from extensive but did indicate the drug treatments we applied provided reasonable blood levels of those antimicrobial drugs. Repeated and more extensive evaluation of blood concentrations over time would be valuable in interpreting future studies. The dosage of amoxicillin we administered might have been suboptimal on the basis of the pharmacokinetics of this antimicrobial drug after oral administration in goats (*34*) and some recent MIC values reported for *B. pseudomallei* (*35*).

A striking observation from this study was that, although treatment with amoxicillin/clavulanate or sulfamethoxazole/trimethoprim had minimal or mild inhibitory effects on abscess formation and the presence of culturable *B. pseudomallei* in tissues, a combination of both treatments appeared highly efficacious. None of the 8 goats treated with all 4 antimicrobial drugs had abscesses visible at necropsy nor had positive organ cultures among the 10 tissues tested. Of interest, antibody titers were lower in goats that received the combination of 4 antimicrobial drugs, suggesting inhibition of infection and a less potent stimulus to the immune system for this treatment group.

In conclusion, our findings indicate PEP with a combination of those 4 antimicrobial drugs might be useful in preventing human cases of melioidosis after exposure to *B. pseudomallei*, and additional studies are justified. In addition, >3 additional features of melioidosis will be necessary to address in future studies with similar models. First, it will be critical to delay the onset of treatment for >48 hours to make the model more realistic for initiation of prophylaxis after later identification of exposure. Second, the animals need to be maintained for longer periods of time after cessation of treatment to determine if prophylaxis does indeed eliminate the infection and prevent recrudescence. Finally, more detailed pharmacokinetic studies should be performed in goats to guide antimicrobial drug dosing. Melioidosis after accidental exposure in the clinical or research laboratory is rare, and it would not be possible to utilize these cases to evaluate the efficacy of PEP. Ultimately, clinical observations in humans are necessary to validate this supposition and to determine the efficacy of PEP in exposed humans.

Appendix Additional information about postexposure antimicrobial drug therapy in goats infected with *Burkholderia pseudomallei*
