# Supplementary material for: Postexposure Antimicrobial Drug Therapy in Goats Infected with Burkholderia pseudomallei
Source: Emerg Infect Dis. 2025 May;31(5):967–75. doi: 10.3201/eid3105.241274 (PMC12044257; doi:10.3201/eid3105.241274)
Supplement: Appendix — Additional information about postexposure antimicrobial drug therapy in goats infected with Burkholderia pseudomallei [file 24-1274-Techapp-s1.pdf]

Article DOI: https://doi.org/10.3201/eid3105.241274

EID cannot ensure accessibility for supplementary materials supplied by authors. Readers who have difficulty accessing supplementary content should contact the authors for assistance.

Postexposure Antimicrobial Drug Therapy in Goats Infected with *Burkholderia pseudomallei*

Appendix.

Appendix Table 1. Clinical time course, euthanasia times and clinical signs.

Treatment	Goat	Euthanasia Day	Clinical Signs at Euthanasia
None	8548	14	Severe
None	8549	14	Mild
Amoxicillin-CA	8432	14	Severe
Amoxicillin-CA	8428	14	Severe
Sulfa-Trimethoprim	8630	14	Severe
Sulfa-Trimethoprim	8631	14	Severe
Sulfa-TM + Amox-CA	8551	14	Severe
Sulfa-TM + Amox-CA	8553	14	Moderate
Amoxicillin-CA	8430	20	Severe
Amoxicillin-CA	8433	20	Severe
None	8628	23	Mild
None	8629	23	Mild
Amoxicillin-CA	8434	23	Severe
Amoxicillin-CA	8429	23	Moderate
Amoxicillin-CA	8431	23	Moderate
Amoxicillin-CA	8435	23	Severe
Sulfa-Trimethoprim	8636	23	Moderate
Sulfa-Trimethoprim	8633	23	Moderate
Sulfa-TM + Amox-CA	8554	23	Moderate
Sulfa-TM + Amox-CA	8556	23	Moderate
None	1198	26	Mild
None	8550	26	Moderate
Sulfa-Trimethroprim	8634	26	Moderate
Sulfa-Trimethoprim	8637	26	Moderate
Sulfa-TM + Amox-CA	8552	26	Moderate
Sulfa-TM + Amox-CA	8436	26	Moderate
None	1199	28	Moderate
None	1197	28	Moderate
Sulfa-Trimethoprim	8632	28	Moderate
Sulfa-Trimethoprim	8635	28	Moderate
Sulfa-TM + Amox-CA	8555	28	Moderate
Sulfa-TM + Amox-CA	8437	28	Moderate

Appendix Table 2. Statistical analyses of differences in outcome by treatment group.

	Treatment Group Comparison*					
**Outcome parameter**	**None vs. AC**	**None vs. ST**	**None vs. AC+ST**	**AC vs. ST**	**AC vs. AC+ST**	**ST vs. AC+ST**
At least one tissue culture positive at necropsy	0.47	0.6	0.007	0.08	0.0002	0.08
Macroabscesses: spleen	1	0.2	0.0002	0.2	0.0002	0.03
Macroabscesses: lungs	0.07	1	0.1	0.03	0.0002	0.1
Macroabsesses: liver	0.2	1	1	0.2	0.2	1
Macroabscesses: kidney	0.2	1	1	0.2	0.2	1
Microabscesses: spleen	0.47	1	0.007	0.2	0.0002	0.03
Microabscesses: lungs	0.01	0.23	0.23	<0.0001	<0.0001	1
Microabsesses: liver	0.57	1	1	0.2	0.2	1
Microabscesses: kidney	1	1	1	1	1	1
Microabscesses: LN	0.2	0.31	0.03	0.007	0.0002	0.47

* None = no treatment, AC = amoxicillin-clavulanate, ST = sulfamethoxazole, AC+ST = combination of both AC and ST. P-values represent results of pairwise comparisons of indicated treatment groups using 2 x 2 contingency tables of proportions of positive and negative animals positive.

Appendix Table 3. Listing of goats by treatment group classified by culture results from tissues at necropsy.

**Treatment**	**Goat**	***B. pseudomallei* confirmed**	**Number of positive tissues of 10 tested**
None	1197	Positive	1
None	1198	Negative	0
None	1199	Positive	1
None	8548	Negative	0
None	8549	Positive	5
None	8550	Positive	3
None	8628	Positive	2
None	8629	Positive	5
Amoxicillin-Clavulanate	8428	Positive	3
Amoxicillin-Clavulanate	8429	Positive	3
Amoxicillin-Clavulanate	8430	Positive	8
Amoxicillin-Clavulanate	8431	Positive	5
Amoxicillin-Clavulanate	8432	Positive	5
Amoxicillin-Clavulanate	8433	Positive	10
Amoxicillin-Clavulanate	8434	Positive	6
Amoxicillin-Clavulanate	8435	Positive	9
Sulfa-Trimethoprim	8631	Negative	0
Sulfa-Trimethoprim	8632	Positive	1
Sulfa-Trimethoprim	8633	Positive	1
Sulfa-Trimethoprim	8634	Positive	3
Sulfa-Trimethoprim	8635	Positive	1
Sulfa-Trimethoprim	8636	Negative	0
Sulfa-Trimethoprim	8637	Negative	0
Sulfa-TM + Amox-CA	8436	Negative	0
Sulfa-TM + Amox-CA	8437	Negative	0
Sulfa-TM + Amox-CA	8551	Negative	0
Sulfa-TM + Amox-CA	8552	Negative	0
Sulfa-TM + Amox-CA	8553	Negative	0
Sulfa-TM + Amox-CA	8555	Negative	0
Sulfa-TM + Amox-CA	8554	Negative	0
Sulfa-TM + Amox-CA	8556	Negative	0

Appendix Table 4. Antibody titers over time post-challenge as determined by the indirect hemagglutination assay.

Treatment	Goat	Day 0	Day 7	Day 14	Day 21
Amoxicillin-Clavulanate	8428	10	160	160	NA
Amoxicillin-Clavulantae	8429	40	80	160	320
Amoxicillin-Clavulanate	8430	40	80	160	NA
Amoxicillin-Clavulanate	8431	40	640	640	2560
Amoxicillin-Clavulanate	8432	40	160	320	NA
Amoxicillin-Clavulanate	8433	80	160	160	NA
Amoxicillin-Clavulanate	8434	80	160	160	320
Amoxicillin-Clavulanate	8435	80	80	640	640
Sulfa-Trimethoprim	8630	40	80	80	NA
Sulfa-Trimethoprim	8631	40	320	1280	NA
Sulfa-Trimethoprim	8632	20	40	80	40
Sulfa-Trimethoprim	8633	40	80	640	1280
Sulfa-Trimethoprim	8634	5*	20	40	320
Sulfa-Trimethoprim	8635	20	160	320	1280
Sulfa-Trimethoprim	8636	40	160	320	160
Sulfa-Trimethoprim	8637	80	160	160	2560
Sulfa+Amoxicillin	8436	80	160	80	40
Sulfa+Amoxicillin	8437	40	80	160	160
Sulfa+Amoxicillin	8551	5*	40	40	NA
Sulfa+Amoxicillin	8552	40	160	320	160
Sulfa+Amoxicillin	8553	40	80	160	NA
Sulfa+Amoxicillin	8554	80	160	160	320
Sulfa+Amoxicillin	8555	10	20	20	20
Sulfa+Amoxicillin	8556	5*	80	160	80
None	8548	80	160	320	NA
None	8628	40	320	1280	5120
None	8629	80	320	640	320
None	8549	40	80	80	NA
None	8550	20	40	80	40
None	1199	80	160	320	NA
None	1198	20	160	320	NA
None	1197	80	320	320	NA
Amoxicillin-Clavulanate	Mean	51	190	300	960
Sulfa-Trimethoprim	Mean	36	128	365	940
Sulfa+Amoxicillin	Mean	31	89	146	148
None	Mean	55	195	420	1827

* Antibody titers <10 were assigned a value of 5 for purposes of calculating means. NA, goats that were euthanized prior to day 21.
